# Isolation and characterization of agro-waste biomass sapodilla seeds as reinforcement in potential polymer composite applications

**DOI:** 10.1016/j.heliyon.2023.e17760

**Published:** 2023-06-28

**Authors:** Nalaeram Sivaram R, Senthil Muthu Kumar Thiagamani, Sivakumar P, Srinivasan M, Surya Narayana Boyina Yagna, Ebrahimnezhad-Khaljiri Hossein, Meena M, Sanjay Mavinkere Rangappa, Suchart Siengchin

**Affiliations:** a Department of Mechanical Engineering, Kalasalingam Academy of Research and Education, Krishnankoil, 626126, Tamil Nadu, India; b Department of Materials Science and Engineering, Faculty of Engineering, University of Zanjan, Zanjan, Iran; c Department of Physics, ST Hindu College, Nagerkoil, 629002, Tamil Nadu, India; d Department of Materials and Production Engineering, The Sirindhorn International Thai-German Graduate School of Engineering (TGGS), King Mongkut’s University of Technology North Bangkok (KMUTNB), Bangkok, Thailand; e Institute of Plant and Wood Chemistry, Technische Universität Dresden, Tharandt, Germany

**Keywords:** Particulate filler, Physio-chemical analysis, Thermal stability, Polymer composites

## Abstract

Fillers or particulate fillers find a growing utilization as reinforcement material in polymer composites due to their ability to enhance the properties of the ensuing composites. The discarded seed in sapodilla fruit is available in abundant and the shell of the seed can be used as a reinforcing filler. The primary goal of this study is to extract and characterize the sapodilla seed shell powder (SSS) physically and chemically in order to assess its potential for reinforcement as a particulate filler in polymer composites. The sapodilla seed shell filler was characterized experimentally by Physio-chemical analysis, X-ray diffraction (XRD), Fourier transform infrared spectroscopy (FTIR) and Energy dispersive X-ray analysis (EDAX). The morphology and the filler size were determined by Scanning electron microscopy (SEM) and Particle size analysis. The thermal degradation behaviour was evaluated by Thermogravimetric analysis (TGA).

## 1 Introduction

Widespread use of polymeric composite materials based on natural reinforcements has become inevitable in almost all applications [[1], [2], [3], [4]]. In recent years natural fibres and particulate fillers are considered as a potential reinforcement material in polymer composites owing to various characteristics such as light in weight, degradable nature and other functional properties which are comparable to their synthetic counterparts [[5], [6], [7], [8], [9], [10]]. In addition, these natural reinforcing materials are cheap and abundantly available [[11], [12], [13], [14], [15], [16]]. The reinforcement of natural fillers in polymers can improve the functional properties of the ensuing composites [[17], [18], [19], [20], [21]]. Selection of appropriate fillers, filler/matrix bonding and suitable fabrication technique can lead to the formation of superior performing composites for potential applications in various industries such as the automotive, aerospace, construction, household, packaging and biomedical etc [[22], [23], [24], [25], [26]].

India is one of the largest agro-based economy that supports numerous global agricultural producing activities. However, there is a shortage of waste management methods, which causes massive amounts of agro-waste to be burned and dumped in landfills. Agro wastes are generated in large quantities every year, yet it is not effectively reused, reprocessed, or discarded [[27], [28], [29], [30], [31]]. One of the effective methods in the management of agro waste is finding ways in the utilization of these wastes as reinforcing fillers in composite applications [[32], [33], [34], [35], [36], [37]]. Several particulate fillers have been extracted from natural resources/agro wastes. To name a few are rice husk [38,39], corn husk [[40], [41], [42]], brewed coffee beans [11,22,43], brewed tea leaves [44,45], banana peels [7,46], tamarind nuts [[47], [48], [49], [50], [51]] and coconut shell [8] etc. Agro-fillers are widely used in polymer composites, because of their good surface finish, better dispersion with the polymer matrix and reasonable physical properties [46]. Furthermore, they also reduce the overall cost of the composites [52,53].

The mechanical characteristics of bio-fillers reinforced composites vary greatly depending upon the physical and chemical characteristics, composition of the material, type of raw fibres or particulates, and growing environment. Recently, a group of researchers extracted and characterized lignocellulosic fillers from *Phaseolus lunatus* and *Vigna radiata* biomass. The cellulose content of the fillers was found to be 65 and 58% respectively. The fillers were thermal stable till a temperature of 333 and 328 °C. The *Phaseolus lunatus* filler possessed around 50% more wax content when compared with the *Vigna radiata* fillers. It is well known that higher the wax content lower is the interfacial bonding between the filler and matrix [54]. Likewise novel natural cellulosic fibers extracted from the stem of manau rattan (Calamus manan) is evaluated for its potential reinforcement in composites. Chemical examination indicated that the manau rattan fiber contained 42% cellulose, 20% hemicellulose, and 27% lignin. The crystallinity index of the manau rattan fiber was determined to be 48.28%, with a crystallite size of 1.91 nm. Thermogravimetric analysis demonstrated that the kinetic activation energy of the manau rattan fibers was 81.68 kJ/mol, and the maximum temperature at which degradation occurred was 332.8 °C. These properties contribute to the manufacturing of composites based on thermoplastic polymers. Further, the manau rattan fibers comprised individual fibers arranged in alignment and were held together by non-cellulosic constituents. Additionally, the surface texture of was found to be coarse, promoting strong adhesion between the fibers and the polymer matrix in composite materials [55]. Similarly, researchers reported that the novel cellulosic fibers from *Abelmoschus Ficulneus* weed possessed 80.6% of cellulose, 36.63% of hemicellulose and the thermal stability was found to be 352.3 °C. From the results it was concluded that these fibers can be a potential reinforcement in polymer composites targeting semi-structural applications. Sunesh et al. [56]extracted and characterized cellulosic micro fillers from *Borassus flabellifer* floret for potential infusion in polymer composites. They reported that the crystallinity index and crystalline size was found to be 69.81% and 70.73 nm respectively. Further, the thermal stability of the filler was found to withstand a temperature up to 200 °C. In another study, modified wood fiber composites bonded with urea formaldehyde (UF) were produced using regenerated tire rubber. The rubber fillers were physically incorporated into the structure of the UF-coated fibers. It was found that the thermomechanical properties of the composites were mainly governed by the wood fibers, whereas the addition of rubber fillers resulted in a significant reduction in moduli and an increase in tan δ values at elevated temperatures, indicating an improvement in damping capability [57]. Similar to this, a large number of novel fibres were isolated, characterized, and used as reinforcement in polymer composites [[58], [59], [60], [61], [62], [63], [64], [65], [66], [67]].

There have been numerous investigations on the fabrication and performance analysis of composites filled with agro fillers. Ojha et al. [68]studied the influence of varying concentrations (5–20 wt%) of wood apple shell (WAS) and coconut shell (CS) on the mechanical properties epoxy composites and found that 15 wt% reinforced WAS epoxy composites exhibited the highest tensile strength of 43.6 MPa while the flexural strength was found to be 78.19 MPa. Similarly, the CS filled (15 wt%) composites displayed a tensile and flexural strength of 41.3 MPa and 68.25 MPa respectively. It is to be noted here that there was a 58% increase in the tensile properties whereas it was 46% increase in the flexural properties. Gokul Kannan et al. [39] investigated the mechanical and thermal properties of agro wastes (coconut shell particulate) filled with polyester matrix. They found that with 3 vol% of coconut shell-filled banana fibre polyester composites, the highest tensile and flexural strengths of 19.91 MPa and 92.761 MPa were attained.

Sapodilla is also known as Manilkara Zapota or naseberry, a long living tree native to the North American continent. It is also grown in large quantities in Asia including India, Malaysia, Thailand and Cambodia. The fruit consists of 3–6 seeds which are hard, glassy and resemble like a bean. The seeds contain hydrocyanic acid which causes headache, dizziness, feeling of suffocation, nausea, etc. When consumed. Hence the seeds should be removed before eating and are thrown as waste. The present study evaluates the potential of sapodilla seed shell (SSS) particulate filler as reinforcement in polymer composites. The evaluation was done based on the Physico-chemical analysis, Fourier Transform Infrared (FTIR) spectroscopy, X-ray diffraction (XRD), Thermogravimetric analysis (TGA), Particle size analysis (PSA), and Scanning Electron Microscopy (SEM).

## 2 Materials and methods

### 2.1 Materials

#### 2.1.1 Sapodilla seeds (SS)

**Fig. 1:**
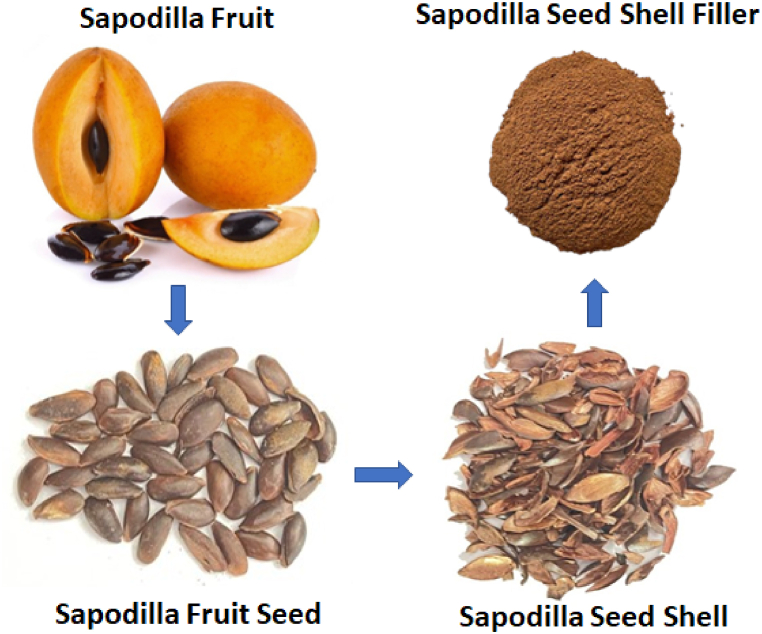
Process of seed collection and the extraction of filler.

Sapodilla fruits were collected from farms in Virudhunagar district of Tamil Nadu, India. For every 20 kg of fruit, 1 kg of seeds were obtained. For further extraction of the filler, the inner kernel of the seed was removed and the outer shell alone was used. The process of seed collection and the extraction of filler is shown in Fig. 1 below.

#### 2.1.2 Extraction of fillers from sapodilla seeds

Initially, the seeds were separated from the fruits and thoroughly cleaned by the water. The seeds were dried under sunlight till the colour of the seeds turn from glassy black to pale brownish colour. Further due to the drying, the bond between the inner kernel and outer shell became weak. Then the outer shell of the seed was peeled off from the inner kernel. The collected seed shells were then ground using a hammer mill machine. The obtained powder was then sieved using various sieve sizes to obtain fine particles of the sapodilla seed shell bio-filler.

### 2.2 Methods

#### 2.2.1 Physio-chemical analysis of the filler

A physio-chemical examination was conducted to identify the chemical constituents such as cellulose, hemicellulose, lignin, wax, ash, moisture, and pectin etc. of the sapodilla filler. The density of the filler was determined using a pycnometer and immersion liquid of distilled water. The volumetric displacement of 1 g of the sample was measured after it was completely immersed in water. The density of the sample was expressed by the weight to volume ratio. The filler’s cellulose content was ascertained using Kurshner and Hofer’s technique. In this the fillers were washed with distilled water and then dried at 80 °C in a oven to eliminate the moisture. 150 mg of the sapodilla seed shell filler was treated with a mixture of ethanol and 95% of nitric acid for about 4 h. The treated filler was again dried in a vacuum oven at a temperature of 60 °C until a constant weight was obtained. The insoluble weight fraction was then calculated by taking in to account the dissolved cellulose. The hemicellulose content in the filler was evaluated in accordance with the NFT 12-008 standard [55]. By treating the samples to mineral acid at high temperatures for 30 min, hemicellulose was measured in the samples. The remaining sample was then heated and mixed with an alkali solution before being dried and weighed. The lignin content of each fraction was calculated using the Klason method. The hydrocarbon-based solvent extraction method of the Conrad method was used to determine the wax content. Ash and moisture content were measured using the IS 199:1989 Indian Standard test method (R2005) [10,40].

#### 2.2.2 Fourier transform infrared (FTIR) spectroscopy

In order to capture the FTIR spectra of the sapodilla filler, a FTIR Spectrophotometer (IR Tracer 100) was used and the spectra was captured in the region of 4000 to 500 cm^−1^ with a scan rate of 32 scans per minute and a resolution of 4 cm^−1^.

#### 2.2.3 X-ray diffraction (XRD)

To determine the crystallinity index of the filler, X-ray diffraction was performed. A spectrometer (BRUKER ECO D8 ADVANCE) was used to conduct the test which produces monochromatic radiation of CuKα with a wavelength of 0.154 nm and recorded at a 2 Theta angle of 10°–80° and at a scan rate of 4°/min. The crystallinity index (CI) and the crystallite size (CS) was evaluated using Equations (1), (2) given below [10,54]:

(1) $$C.I=\frac{I002-IAM}{IAM}\times 100$$

where I_002_ refers to the maximum intensity of the crystalline materials and I_AM_ refers to the diffraction rate of amorphous materials.

(2) $$CS=\frac{K\lambda }{\beta \;\mathrm{Cos}\;\theta }\times 100$$

where K is the Scherrer constant (0.94), λ is the wavelength of the x-ray beam (0.154 nm), and β is the peak full width half maximum (FWHM).

#### 2.2.4 Particle size analysis

A Shimadzu SALD-2300 (wingSALD II: version 3.1.1) particle size analyser was used to measure the extracted filler’s particle diameter. Every 100 particles were divided into ranges, and the particle size was studied.

#### 2.2.5 Scanning electron microscopy (SEM)

A scanning electron microscope (Carl Zeiss) was used to record the filler’s morphology. The fillers were scanned using several electron wavelengths with magnifications ranging from 500 to 10 K with an accelerated voltage of 20 kV.

#### 2.2.6 Thermogravimetric analysis (TGA)

A thermogravimetric analyser (STA6000, Perkin Elmer, USA) was used to test the extracted filler’s thermal stability. The thermograms were recorded at a heating rate of 20 °C/min throughout a temperature range of 30–750 °C. A nitrogen environment with a flow rate of 60 ml/min was used for the test.

## 3 Results and discussions

### 3.1 Physio-chemical properties

The weight of composites is influenced by the filler density. The filler under investigation in the current study was determined to have a density of 0.839 g/cm^3^. The chemical composition of the fillers has a significant impact on the performance of the composites [10,54]. The origin of the plant has a significant impact on the chemical components of the plant-based filler. The chemical composition of the sapodilla filler compared with other fillers reported earlier is depicted in Table 1.

**Table 1:** Physicochemical composition of Sapodilla seed filler.

Name of the filler	Density (g/cc)	Cellulose (%)	Hemi cellulose (%)	Lignin (%)	Pectin (%)	Wax (%)	Ash (%)	Moisture (%)	Ref.
Sapodilla Seed Shell filler	0.839	43.94	20.93	15.65	3.58	1.34	1.26	13.43	Present work
Phaseolus lunatus	0.46	65.2	20.1	11.3	6.7	1.13	7.2	7.2	[54]
Vigna radiata	0.53	58.2	21.9	16.4	8.3	0.6	7.6	7.6	
Tamarind seed	0.48	19.22	47.5	18.8	–	–	–	–	[73]
Date palm seed	–	20	55	23	–	–	1.1	–	[74]

The sapodilla seed shell powder is majorly comprised of three major constituents such as cellulose, hemicellulose and lignin. The cellulose is composed of long polymer chains of glucose arranged in an orderly fashion and display a crystalline structure [69,70]. Furthermore, the hydroxyl groups of glucose moieties of cellulose chains involve in the inter-molecular hydrogen bonding. Contrary, the hemicellulose consists of random polymer chains which are rich in branches and as a result exhibit amorphous structure. The cellulose, hemicellulose, lignin and pectin content of the filler was found to be 43.94, 20.93, 15.65 and 3.58% respectively. Similarly, the wax, ash and moisture content were recorded as 1.34, 1.26 and 13.43%. The OH groups in cellulose result in a great deal of intra- and intermolecular hydrogen bonding between the hydrogen and oxygen molecules. As a result, these forces effectively hold the cellulose chains and hence leads to enhanced properties. Furthermore, due to presence of potentially reactive methylol and phenolic OH groups which makes the filler have better binding with the polymer chains [71]. In contrast, increased hemicellulose concentrations may have detrimental impacts on mechanical qualities. The capacity of the fibre to bind with the matrix during the production of composites decreases as wax and moisture contents rise. It is to be noted that generally fillers extracted from fruits would have higher lignin content [72]. Same trend could also be found in case of the sapodilla seed shell filler.

### 3.2 FTIR analysis

**Fig. 2:**
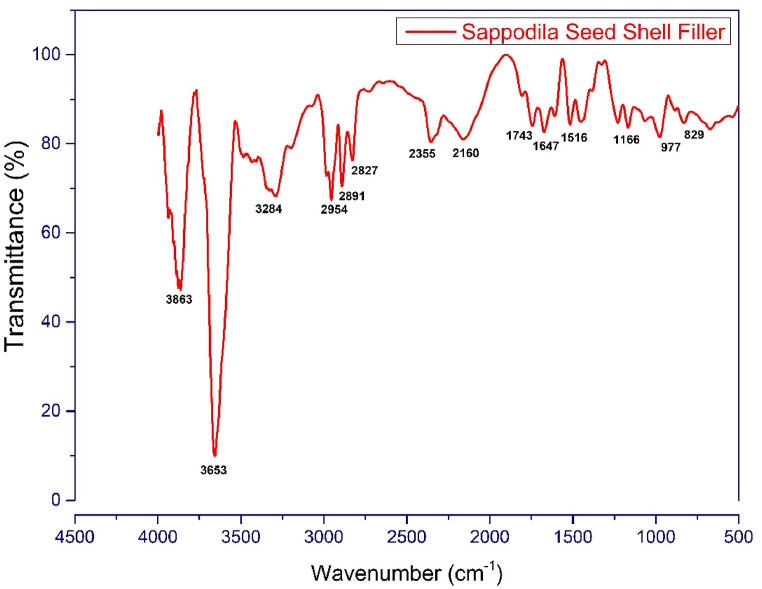
FTIR spectrum of the sapodilla seed filler.

The FT-IR test has been used as a technique for the elucidation of chemical structure of sapodilla seed shell. The FT-IR spectra of the sapodilla seed filler is presented in Fig. 2. From the FT-IR spectra, the absorption peaks of stretching OH at 3863 and 3653 cm^−1^ were seen, which can be attributed to the cellulose, hemi-cellulose or lignin components [75,76]. The peak of N–H at 3284 cm^−1^ was observed [77]. The depicted peak at 2954 cm^−1^ can be due to alkyl (C–H) stretch [78]. The appeared peak at 2891 cm^−1^ belongs to C–H stretching in aldehyde group [–(O=)C–H], which can be sign of cellulose, hemi-cellulose and lignin in sapodilla seed shell [79]. Vibration stretch CH_3_ group was appeared at peak of 2827 cm^−1^. The band at 2355 cm^−1^ represents the C≡C stretching of wax in the sapodilla seed shell [80]. The peak around the 2160 cm^−1^ can be due to stretching of alkenyl (C–H and C=C stretch). The peak at 1743 cm^−1^ ascribes the carbonyl group stretching of ester groups in pectin [81]. The peak corresponds to 1674 cm^−1^ represents C=O peak in lignin. Also, the peak around 1516 cm^−1^ can be due to benzene ring vibration of lignin. The presents of cellulose and hemi-cellulose can be confirmed from the peak at the near 1452 cm^−1^. This peak shows the symmetric deformation of methylene group (CH_2_) of cellulose [82]. The absorbance peak at 1228 cm^−1^ corresponds to the CH stretching vibration of acetyl group in lignin [83]. The apparent peak around the 1166 cm^−1^ can be attributed to C–O–C stretching in ether of 1–3 linked xyloglucan of hemi-cellulose [79]. Band at 977 cm^−1^ shows the C–H bending of alkene [84]. The bending vibration of C–H in lignin appeared as band at 829 cm^−1^ [79].

### 3.3 XRD analysis

**Fig. 3:**
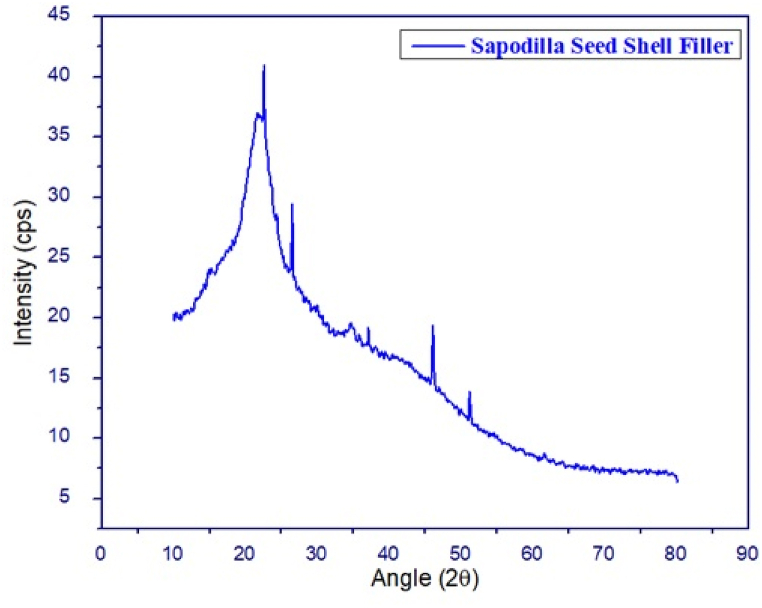
X-ray diffractograms of the sapodilla seed filler.

Fig. 3 depicts the XRD diffractogram of sapodilla seed shell. According to the other literatures [85] cellulose has crystalline nature, whereas, the lignin shows the amorphous behaviour. For this reason, the characterized peaks can be related to various cellulose structures into the sapodilla seed shell. The characterized peak at 2θ around the 22.5° can be attributed to (200) plane of cellulose I [86]. Also, the peak corresponds to 2θ around 26.47° can present (002) plane of cellulose I [87]. The depicted peak at 2θ = 37.07° can be corresponds to (040) crystallographic plane of cellulose II [88]. The peaks around 46.05° and 51.17° may correspond to the crystal structure of cellulose [89]. The crystallinity index calculated as per equation (1) was found to 31.11% and the average crystallite size was found to be 41.91 nm. The crystallite size corresponding to the peak angle is presented in Table 2.

**Table 2:** Crystallite size of the sapodilla seed filler.

Peak (2θ)	Crystallite size (nm)
21.03	39.31
22.52	55.43
26.46	22.60
29.95	19.54
46.05	51.67
51.17	62.91
**Average size**	**41.91 nm**

### 3.4 Particle size analysis

**Fig. 4:**
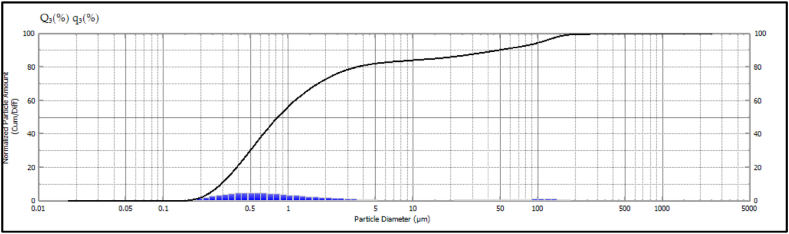
Particle size analysis of the sapodilla seed filler.

Fillers could be used to reduce the costs and to provide strength. Strength imparting properties are greatly influenced by the particle size and by the surface chemistry. The particle size of the fillers reported have particle sizes varying from few nano meters to micrometres. In order to determine the particle size of the sapodilla fillers, the particle size analysis was performed and the results are presented in Fig. 4. From the results it is evident that the average particle size of the fillers was found to be 1.46 μm. However, majoring of the particles lied in the range of 0.2–1 μm.

### 3.5 Morphological analysis

**Fig. 5:**
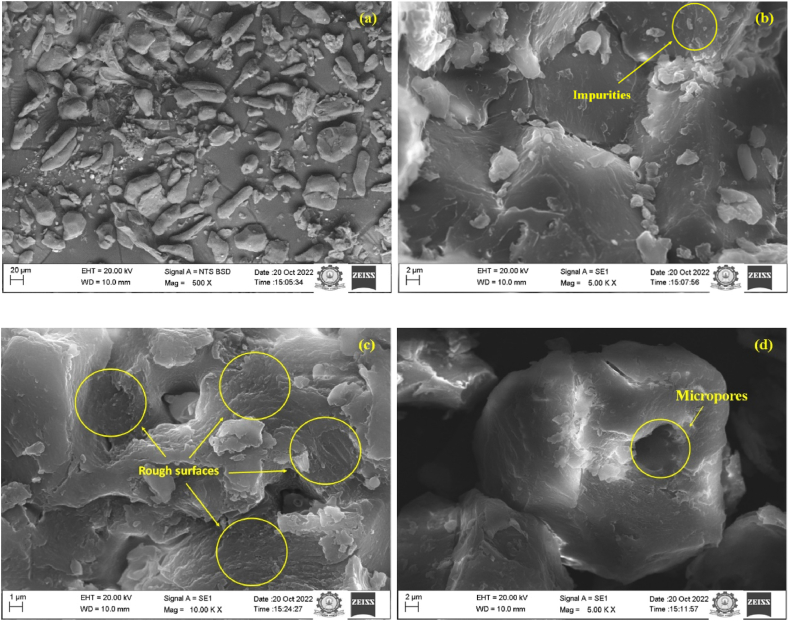
SEM micrographs of the sapodilla seed filler.

In order to investigate the morphology of the sapodilla seed shell filler scanning electron microscopy (SEM) was performed and are presented in Fig. 5. From the SEM images it could be ascertained that the fillers have an irregular shape (Fig. 5a). From Fig. 5b it could be seen that some extra cellular impurities are present in the filler surface. The surface of the filler is found to be distinctly uneven with rough irregular surfaces (Fig. 5c). Furthermore, the filler also possessed some micropores (Fig. 5d). The presence of micropores could assist in the filler matrix bonding when blended with a polymer to form polymer composites [43,54].

### 3.6 EDAX analysis

**Fig. 6:**
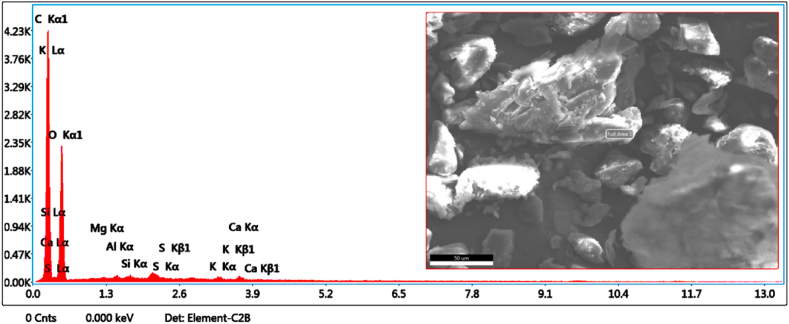
EDAX spectra of the sapodilla seed filler.

Presence of calcium, potassium and iron ion was proved with EDAX spectra depicted in Fig. 6. High energy peak at 3.9 keV which points out the richness of K than Ca in the seed shell. As in photosynthesis process, potassium ion played a vital role in opening and closing of the guard cells, in such a way presence of K ions, may control the pores in polymer surface when it dispersed in the polymer.

### 3.7 Thermogravimetric analysis

**Fig. 7:**
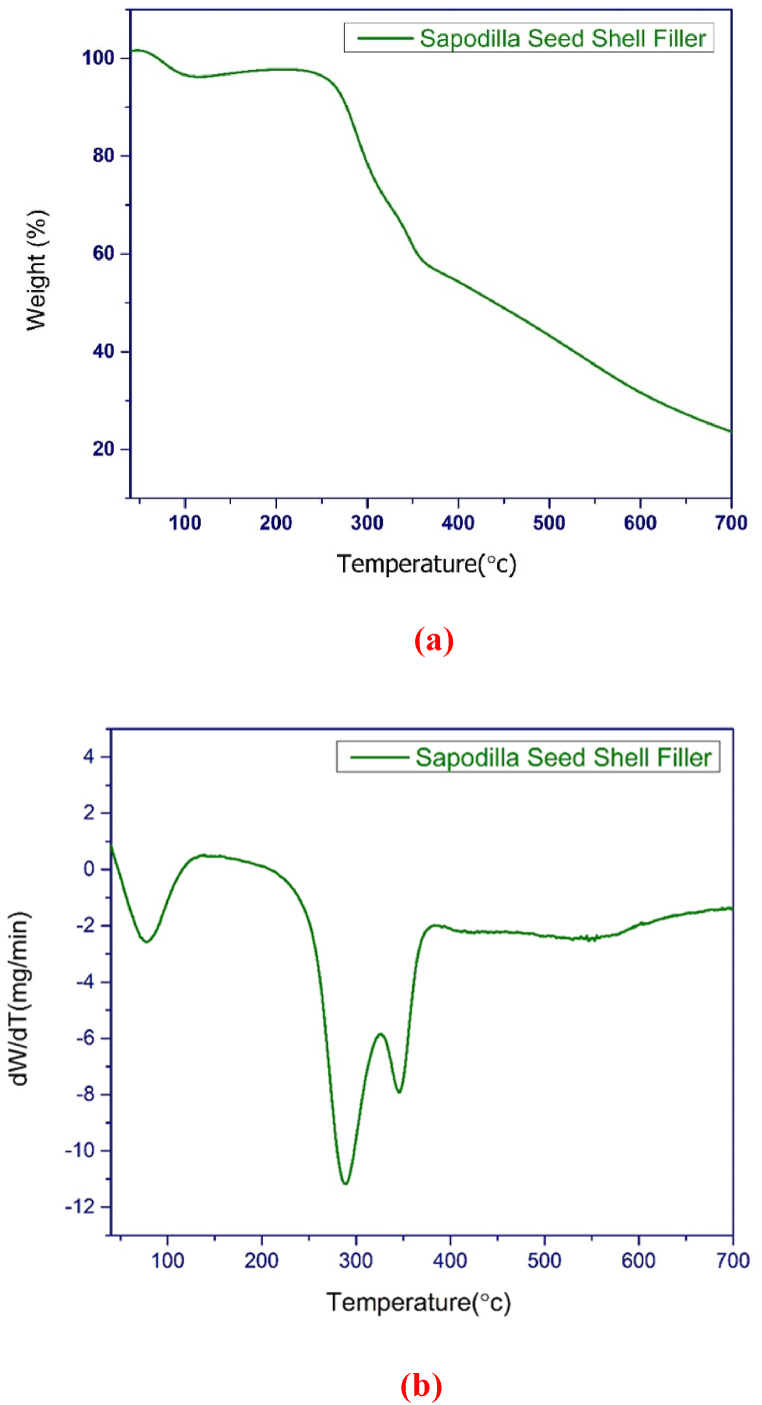
(a) Primary thermograms of the sapodilla seed filler; (b) Derivative thermograms of the sapodilla seed filler.

The thermal stability of sapodilla seed shell can be analyzed by the primary and derivative thermograms as obtained from the thermogravimetric analysis. As can be seen in Fig. 7(a and b) the profile depicts four stage degradation.

The inflection temperatures related to the maximum rate of degradation with respect to the different stages are present in Table 3. In the temperature of 38–118 °C, the first weight loss occurred due to evaporation of moisture. The weight loss in this stage was about 3.75%. In the second stage, the pyrolysis of hemicellulose happened at the temperature range of 200–318 °C. The measured weight loss at this stage was about 28.2%. The third stage was seen at the temperature range of 330–370 °C, which can be massive degradation of cellulose (about 42.75% weight loss). After this stage, the slow degradation of lignin was occurred at the temperature range of 440–600 °C, which caused to loss about 68.3% of initial weight. The char residue at the temperature of 734 °C was about 21.36%.

**Table 3:** Inflection temperatures related to the maximum rate of degradation.

Degradation temperature (°C)	Sapodilla seed shell filler				
	Stages	Onset	Inflection	End set	Residue (%)
	**Stage 1**	51.12	76.98	103.32	21.36
	**Stage 2**	255.56	288.49	302.65	
	**Stage 3**	338.59	345.06	359.62	
	**Stage 4**	535.62	548.39	595.22	

## 4 Conclusions

For the first time the agro-waste in the form of sapodilla seed shell has been extracted and characterized to assess its potential to be a reinforcing filler in polymer composites for various applications. Physio-chemical analysis revealed that the filler consists of 43.94% cellulose, 20.93% hemicellulose, 15.65% lignin, 3.58% pectin, 1.34% wax and 1.26% ash contents. The density of the filler was found to be 0.839 g/cc. The particle size analysis revealed that the average particle size of the fillers was found to be 1.46 μm. However, majority of the particles lied in the range of 0.2–1 μm. Form the morphological analysis it was evident that the surface of the filler is found to be distinctly uneven with rough irregular surfaces and also possessed some micropores. The EDAX spectrum revealed the presence of K ions in the filler which may control the pores in polymer surface when it dispersed in the polymer. The thermal degradation of the filler took place in four stages where the maximum degradation took place at the temperature of around 330 °C. Hence, based on the results obtained, the sapodilla seed shell filler could be considered as a potential reinforcement material in polymer composites.

## Author contribution statement

Nalaeram Sivaram R, Senthil Muthu Kumar Thiagamani: Conceived and designed the experiments; Performed the experiments; Analyzed and interpreted the data; Contributed reagents, materials, analysis tools or data; Wrote the paper.

Sivakumar P, Srinivasan M, Boyina Yagna Surya Narayana, Hossein Ebrahimnezhad-Khaljiri, Meena M, Sanjay Mavinkere Rangappa, Suchart Siengchin: Conceived and designed the experiments; Analyzed and interpreted the data; Wrote the paper.

## Funding

Nil.

## Data availability statement

Data included in article/supp. material/referenced in the article.

## Additional information

No additional information is available for this paper.

## Declaration of competing interest

The authors declare that they have no known competing financial interests or personal relationships that could have appeared to influence the work reported in this paper
